# A Paper-Based Analytical Device Integrated with Smartphone: Fluorescent and Colorimetric Dual-Mode Detection of β-Glucosidase Activity

**DOI:** 10.3390/bios12100893

**Published:** 2022-10-18

**Authors:** Wei-Yi Zhang, Tao Tian, Li-Jing Peng, Hang-Yu Zhou, Hao Zhang, Hua Chen, Feng-Qing Yang

**Affiliations:** 1 Chongqing Key Laboratory of High Active Traditional Chinese Drug Delivery System, Chongqing Medical and Pharmaceutical College, Chongqing 401331, China; 2 School of Chemistry and Chemical Engineering, Chongqing University, Chongqing 401331, China

**Keywords:** paper-based analytical device, fluorescence, colorimetric, dual-mode smartphone sensor, β-glucosidase activity

## Abstract

In this work, indoxyl-glucoside was used as the substrate to develop a cost-effective, paper-based analytical device for the fluorescent and colorimetric dual-mode detection of β-glucosidase activity through a smartphone. The β-glucosidase can hydrolyze the colorless substrate indoxyl-glucoside to release indoxyl, which will be self-oxidized to generate green products in the presence of oxygen. Meanwhile, the green products emit bright blue-green fluorescence under ultraviolet–visible light irradiation at 365 nm. Fluorescent or colorimetric images were obtained by a smartphone, and the red-green-blue channels were analyzed by the Adobe Photoshop to quantify the β-glucosidase activity. Under the optimum conditions, the relative fluorescent and colorimetric signals have a good linear relationship with the activity of β-glucosidase, in the range of 0.01–1.00 U/mL and 0.25–5.00 U/mL, and the limits of detection are 0.005 U/mL and 0.0668 U/mL, respectively. The activities of β-glucosidase in a crude almond sample measured by the fluorescent and colorimetric methods were 23.62 ± 0.53 U/mL and 23.86 ± 0.25 U/mL, respectively. In addition, the spiked recoveries of normal human serum and crude almond samples were between 87.5% and 118.0%. In short, the paper-based device, combined with a smartphone, can provide a simple, environmentally friendly, and low-cost method for the fluorescent and colorimetric dual-mode detection of β-glucosidase activity.

## 1. Introduction

β-Glucosidase (β-D-glucoside glucohydrolase, EC 3.2.1.21) is a hydrolase that can catalyze the hydrolysis of β-D-glycosidic bonds of carbohydrate and glucose derivatives to obtain β-D-glucose and corresponding aglycones [1]. β-Glucosidase widely exists in animals, plants, bacteria, fungi, yeast, and other life systems [2], which involves the secondary metabolism, biomass conversion, activation of plant defense system, and regulation of plant hormones and closely relates to the physiological functions of organisms [3,4]. In the human body, β-glucosidase, which acts as a lysosomal enzyme, participates in the formation of ceramide [5]. The gene that controls the activity of β-glucosidase is confirmed to be the causative gene of Gaucher’s disease, which is also related to the pathogenesis of Parkinson’s disease [6,7]. Moreover, β-glucosidase in serum is a biomarker for the diagnosis of necrotizing enterocolitis, and malignant cells also contain more β-glucosidase than normal cells [8,9]. In the food industry, β-glucosidase can act as a flavor enzyme to enhance the aroma of juice and wine [10]. It is also used to hydrolyze isoflavone glycosides to isoflavone aglycones in soybean products that are more conducive to intestinal absorption [11]. In addition, monitoring the activity of β-glucosidase can reveal the hydrolysis mechanism of the active ingredients in *Chrysanthemum morifolium*, Semen Armeniacae Amarum, Radix Scrophulariae, and other plant materials during processing and storage [12]. In agriculture, β-glucosidase is involved in the degradation of agricultural waste, and its activity is one of the important indicators used for evaluating the soil quality [13,14]. In environmental studies, the activity of β-glucosidase in lake water is closely related to the decomposition of phytoplankton bloom during the spring [15]. Due to the important roles of β-glucosidase in medical diagnosis, food industry, and agricultural and environmental monitoring, it is necessary to develop simple and reliable methods to detect its activity.

In the traditional method for detecting β-glucosidase, the substrate *p*-nitrophenyl-β-D-glucopyranoside was hydrolyzed to *p*-nitrophenol, and the activity of β-glucosidase was detected by measuring the characteristic absorption peak of *p*-nitrophenol at 405 nm [11]. However, the substrate of this method is very unstable, and environmentally hazardous reagents are required [16]. In recent years, fluorescence-based methods have frequently been used in the analytical field, due to their high sensitivity, fast response, and easy use. Several fluorescent probes have been designed for the detection of β-glucosidase activity [17,18,19]. However, these fluorescence-based methods require a complex synthesis process, large-scale detection instruments, and professional operators. Previously, a personal glucose meter (PGM) method was developed (by us) to detect β-glucosidase activity [20]. In the study, β-glucosidase catalyzes the D-(−)-salicin to produce glucose and saligenin. Glucose and saligenin react with glucose dehydrogenase and K_3_[Fe(CN)_6_] on the glucose test strip, respectively, resulting in a PGM detectable signal. Although the PGM method can realize the point-of-care testing (POCT) of β-glucosidase activity, there is the problem of the high detection limit (450 U/L). In addition, these existing methods are based on the single-mode detection of β-glucosidase activity, which is susceptible to external interference, such as complex biological environment and different operators [21]. In contrast, the dual-mode analysis method can produce two kinds of detection signals, which can mutually verify the determination results [22]. The dual-mode analysis method has the characteristics that each sensing mode can meet the detection requirements under different detection conditions [23]. Therefore, the multiple detection mode is beneficial for improving the reliability and sensitivity of the detection method. For example, Yu et al. developed a colorimetric and electrochemical dual-mode method for the detection of hydrogen peroxide (H_2_O_2_), based on the peroxidase-like activity and excellent electrocatalytic H_2_O_2_ reduction activity of MOF-818 [24]. On the basis of the “signal turn-off” caused by the destruction of MOF-818 enzyme-like activity and electrochemical catalytic activity by hydrogen sulfide (H_2_S), the method was further used for the colorimetric and electrochemical dual-mode detection of H_2_S. By the reaction of *p*-aminophenol (AP) and N-[3-(trimethoxysilyl)propyl]ethylenediamine, Chen et al. synthesized the silicon nanoparticles (Si NPs) with both fluorescent and colorimetric signals [21]. Because AP can be produced through catalyzing the 4-aminophenol phosphate by alkaline phosphatase (ALP), and the intensity of the two signals of Si NPs are related to the concentration of AP, a fluorescent and colorimetric dual-mode detection method was developed to determine the ALP activity. Combining the method with enzyme-linked immunosorbent assay, a fluorescent and colorimetric dual-mode detection platform for human prostate-specific antigen was further constructed.

Since Müller and Clegg first constructed the paper-based microfluidic channel in 1949 and Whitesides et al. first used the microfluidic paper-based analytical device (μPAD) as the detection platform in 2007, the paper-based device has a significant impact on the academia and industry with its unique advantages [25,26]. As a biodegradable natural material, cellulose paper, the base material of paper-based device, has the characteristics of being abundant, lightweight, and inexpensive [27]. The network structure of cellulose provides a large specific surface area and makes the liquid flow through capillary action, based on its hydrophilicity and porosity [28]. By means of wax printing, inkjet printing, cutting, chemical vapor deposition, photolithography, flexography, and screen printing, a guide channel can be constructed on the paper to realize the controllability of liquid flow [29]. The μPAD is considered to be a promising diagnostic tool for developing countries, due to its low price and user-friendliness, by the World Health Organization [30]. According to the different sensing principles of μPADs, some portable analytical tools are used to combine with μPADs to develop low-cost POCT devices. For example, the combination of a vernier caliper and a μPAD, based on the viscosity change, can develop a visual distance readout analytical device, and the combination of a portable scanner and a μPAD, based on color change, can develop a colorimetric analytical device [30,31,32,33]. Among portable analytical tools, the smartphone has gained the most extensive applications, due to its powerful analytical capabilities and high popularization rate. The smartphone is equipped with cameras, ambient light sensors, gyroscopes, and other sensing accessories, with high optical sensing and imaging performance [34]. In addition, the smartphone can transmit image data through Bluetooth or image sharing for remote monitoring and connect with the cellular mobile network, as well as combine with image analysis applications, to achieve the real-time processing of image data [35]. Different analytical platforms integrating μPADs and smartphones have been reported. For example, Han et al. developed a paper-based, lateral-flow sensor using a smartphone to record the watermark coverage area on the pH test paper of the test solution [36]. A paper-based colorimetric sensor using a smartphone to record the color change of paper-based devices was previously developed by us [37]. In addition, Zhou et al. developed a paper-based fluorescent sensor using a smartphone to record the paper-based fluorescence changes [38].

**Figure 1:**
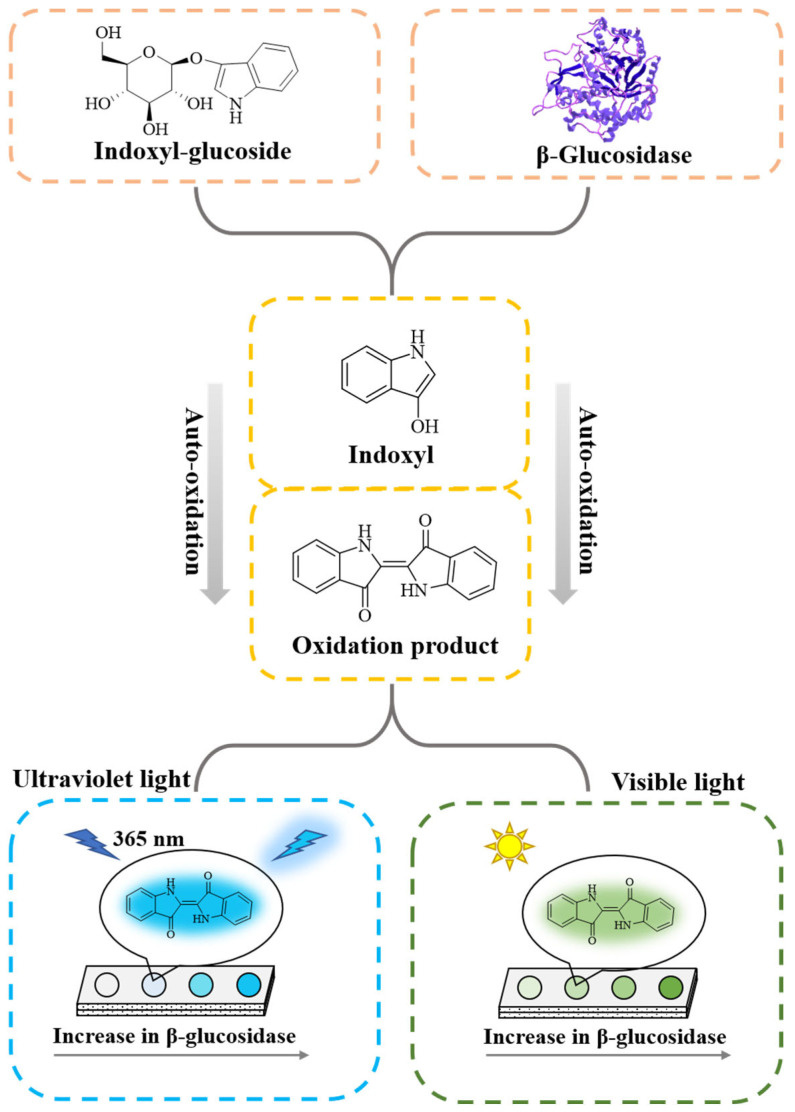
Schematic illustration of fluorescent and colorimetric dual-mode detection of β-glucosidase, based on the paper-based device using indoxyl-glucoside as a substrate.

In this work, a paper-based analytical device, combined with a smartphone to realize the fluorescent and colorimetric dual-mode detection of β-glucosidase activity, was constructed. The detection principle is shown in Figure 1. β-Glucosidase can hydrolyze the β-D-glycosidic bond of substrate indoxyl-glucoside and release indoxyl, which is easily oxidized to form green oxidation product in the presence of oxygen, emitting blue-green fluorescence under UV irradiation at 365 nm. Therefore, the higher the β-glucosidase activity, the greater the intensity of the fluorescent and colorimetric signals of the oxidation products. The fluorescent or colorimetric images of the paper-based device are digitally recorded in pixels by a smartphone. The red-green-blue (RGB) channel value of each pixel in the image can be obtained through Adobe Photoshop by analyzing the color layer of the image. Thus, the fluorescent or colorimetric signal of the image can be transformed into quantifiable data points. Finally, the constructed paper-based device was optimized and applied in the detection of β-glucosidase activity in normal human serum and crude almond samples.

## 2. Materials and Methods

### 2.1. Materials and Reagents

Indoxyl-glucoside, β-glucosidase (from almonds, 12 U/mg), D-(+)-glucose, α-glucosidase (25.4 U/mg), and xanthine oxidase (XOD, 10.6 U/mg) were purchased from Shanghai Yuanye Bio-Technology Co., Ltd., (Shanghai, China). Urease (200–300 U/mg) was purchased from Shanghai Macklin Biochemical Co., Ltd., (Shanghai, China). Sodium dihydrogen phosphate, phosphoric acid, potassium chloride, sodium chloride, L-(+)-ascorbic acid, and magnesium sulfate heptahydrate were purchased from Chengdu Chron Chemicals Co., Ltd., (Chengdu, China). Glutathione was purchased from Shanghai Adamas Reagent Co., Ltd., (Shanghai, China). Sodium hydroxide was purchased from Titan Scientific Co., Ltd., (Shanghai, China). Bovine serum albumin (BSA) was purchased from Sangon Biotech (Shanghai) Co., Ltd., (Shanghai, China). Calcium chloride anhydrous was purchased from Tianjin Damao Chemical Reagent Factory (Tianjin, China). L-Serine was purchased from Tianjin Guangfu Fine Chemical Research Institute (General Partnership) (Tianjin, China). L-Histidine was purchased from Chengdu Huaxia Chemical Reagent Co., Ltd., (Chengdu, China). Crude almond was purchased from Anhui Bozhou Yiletang Business Co., Ltd., (Bozhou, China). The normal human serum was purchased from Beijing Solarbio Science and Technology Co., Ltd., (Beijing, China). Whatman grade 1 filter paper was purchased from GE (China) Co., Ltd., (Shanghai, China). PVC adhesive pad was purchased from Shanghai Jieyi Biotechnology Co., Ltd., (Shanghai, China). All the solutions were prepared in ultrapure water.

### 2.2. Instrumentation

**Figure 2:**
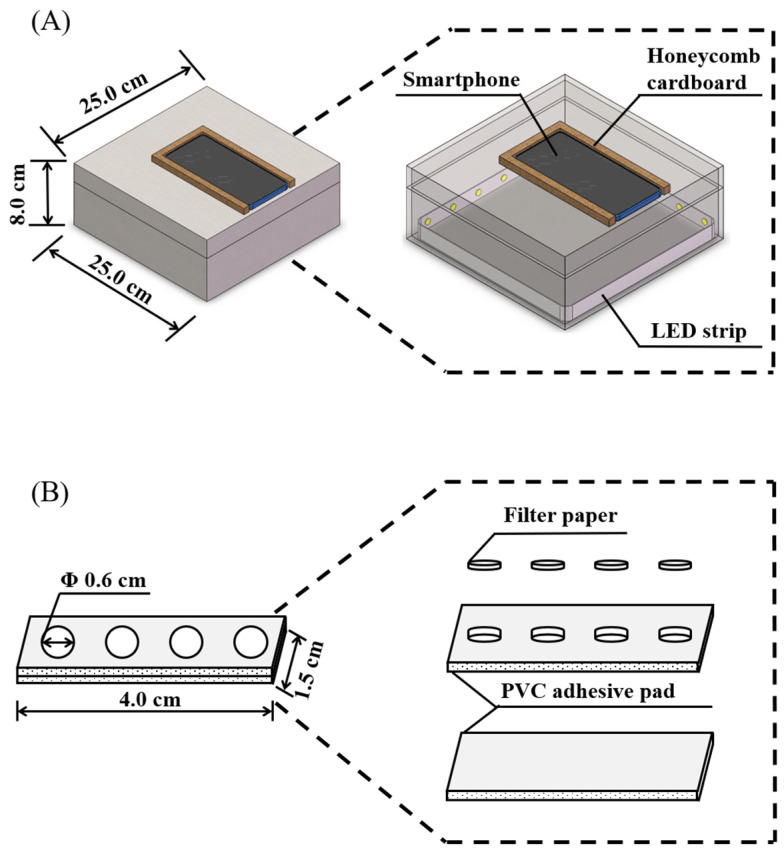
Structure diagram of homemade camera obscura (**A**) and paper-based device (**B**).

A DHG-9015A air blast drying oven (Shanghai Yiheng Technology Instrument Co., Ltd., Shanghai, China) was used for drying paper-based device. A DZF-6012 vacuum drying oven (Shanghai Yiheng Technology Instrument Co., Ltd., Shanghai, China) was used to control the temperature during the enzymatic reaction. An ATSelem 1820A water-purification apparatus (Chongqing Antesheng Environment-Protection Equipment Co., Ltd., Chongqing, China) was used to prepare the ultrapure water used in the experiments. A TGL-20 M high-speed refrigerated centrifuge (Hunan Xiangxin Instrument and Meter Co., Ltd., Changsha, China) was used to centrifuge crude almond sample. A Redmi K40 smartphone was used to shoot fluorescence and colorimetric images. A homemade camera obscura was combined with the smartphone to obtain colorimetric images. The fabrication method of the camera obscura is similar to the previous work [39,40]. The structure of the camera obscura is shown in Figure 2A: a small opening at the top of the camera obscura to expose the phone camera; honeycomb cardboard strips were affixed around the location of the smartphone to limit the location; the LED strip (12 W/m) inside the camera obscura provided a stable and uniform light source. For each shot, the paper-based device was placed in the same position at the bottom of the camera obscura. A ZF-1 ultraviolet analyzer (Shanghai Lichen Bangxi Instrument Technology Co., Ltd., Shanghai, China) provides UV light source and is combined with the smartphone to obtain fluorescent images.

### 2.3. The Fabrication of Paper-Based Analytical Device

The fabrication method of the paper-based device is similar to our previous work [33,37]. Its structure is divided into three parts: a complete PVC adhesive pad; a PVC adhesive pad with small holes (pore size is 6.0 mm); and the filter paper wafers with the diameter of 6.0 mm. The complete and perforated PVC adhesive pad were glued together (the touchable area), and the filter paper wafers were embedded in the round hole of the PVC adhesive pad (the detection area), which constitutes the paper-based device (Figure 2B). Then, 5.0 μL of indoxyl-glucoside, with a concentration of 7.0 mM, was dropped onto the filter paper of the paper-based device, and the surface was dried at 35 °C for 5.0 min to obtain the final paper-based device.

### 2.4. Fluorescent and Colorimetric Dual-Mode Detection of β-Glucosidase Activity by the Paper-Based Device

The 10.0 μL of different concentrations of β-glucosidase solution were added to the detection area of the paper-based device, and then reacted for 30.0 min at 30 °C. For fluorescent detection, under irradiation at 365 nm, the fluorescent image of paper-based device was captured by the smartphone. For colorimetric detection, the LED strip in the homemade camera obscura provides light source, and the colorimetric image of the paper-based device was captured by the smartphone. The fluorescent and colorimetric images were processed by the Adobe Photoshop CC 2018, and the color intensity of the RGB channel of the image was analyzed to reflect the fluorescent or color change of the image. Finally, the fluorescent or colorimetric signal (y) and the relative fluorescent or colorimetric signal (Δy) of the detection area of the paper-based device were calculated according to Equations (1) and (2), respectively.

(1) $$y=\left(Green-Red\right)\;value$$

(2) $$\;\;\;\Delta y=y_{test\;sample}-y_{blank\;sample}$$

### 2.5. β-Glucosidase Assay in Real Samples

The prepared paper-based device was used to detect the β-glucosidase activity in crude almond and human serum samples to evaluate its practical application ability. The crude almonds were crushed into powder by an RHP-600 high-speed multifunctional crusher (Zhejiang RongHao Industry & Trade Co., Ltd., Jinghua, China). A total of 2.0 g of crude almond powder was mixed with 10.0 mL of ultrapure water, and then ground to homogenate in an ice bath. The homogenate was centrifuged at 4361× *g* for 10.0 min, and the process was repeated twice to remove solid impurities. The obtained solution was filtered through a 0.22 μm nylon membrane filter (Anpel Laboratory Technologies (Shanghai) Inc., Shanghai, China) three times to obtain a clear and transparent crude almond sample, which was diluted by ultrapure water (64 times for fluorescent detection and 32 times for colorimetric detection) to meet the linear range of the developed method, and an equal volume of different activity of β-glucosidase solution was added to obtain the spiked crude almond sample solutions. Human serum sample without other pretreatment was diluted 200 times with ultrapure water, and then an equal volume of different activity of β-glucosidase solution was added to prepare the spiked human serum sample solutions. Then, 10.0 μL of spiked sample solution was added to the detection area of the paper-based device and reacted at 30 °C for 30.0 min. The activity of β-glucosidase in the actual samples were calculated by the linear relationship between the activity of β-glucosidase and the relative fluorescent or relative colorimetric signal. Calculation of standard recovery of actual samples was performed by Equation (3).

(3) $$\mathrm{Recovery}\;\mathrm{rate}\;(\%)=\frac{\;\mathrm{Measured}\;\mathrm{activity}\;-\;\mathrm{Initial}\;\mathrm{activity}}{\mathrm{Added}\;\mathrm{activity}}\;\times \;100$$

## 3. Results and Discussion

### 3.1. Sensing Mechanism

**Figure 3:**
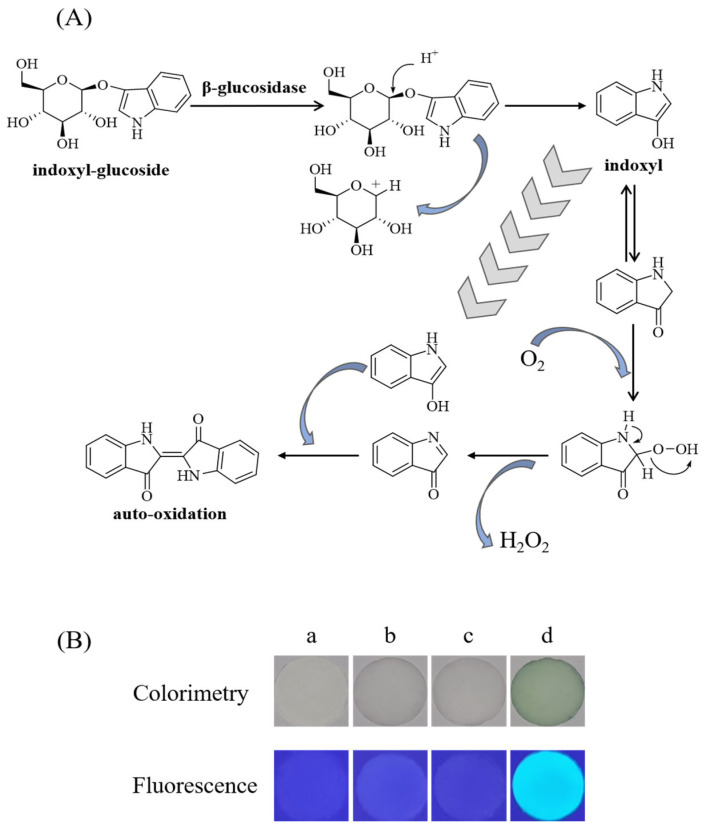
(**A**) Reaction mechanism of indoxyl-glucoside in the presence of β-glucosidase. (**B**) Fluorescent and colorimetric diagrams of paper-based device in the presence of different reagents: (**a**) pure filter paper, (**b**) indoxyl-glucoside, (**c**) β-glucosidase, (**d**) indoxyl-glucoside and β-glucosidase.

The detection principle of this study is based on the specific reaction between substrate indoxyl-glucoside and β-glucosidase (Figure 3A) [41]. The colorless substrate indoxyl-glucoside can be catalyzed to release colorless aglycone indoxyl by the β-glucosidase. Indoxyl undergoes auto-oxidation in the presence of oxygen and can form green products. Moreover, the substrate indoxyl-glucoside does not emit fluorescence under UV irradiation at 365 nm, while the first phase product indoxyl and the second phase of the indoxyl self-oxidation product can emit blue-green fluorescence under irradiation at 365 nm. According to this principle, the fluorescent and colorimetric dual-mode detection of β-glucosidase may be realized.

However, the indoxyl auto-oxidation product is a colored precipitate, which is not suitable for the reaction in solution, and the paper-based device provides an alternative option. Combined with a smartphone, it is expected to develop a simple method for detecting the β-glucosidase activity. As shown in Figure 3B(a), the white filter paper does not emit fluorescence under irradiation at 365 nm. The addition of indoxyl-glucoside or β-glucosidase solution to the filter paper does not significantly change the color and the fluorescent property of the filter paper (Figure 3B(b–c)). When indoxyl-glucoside and β-glucosidase were added together to the filter paper, the color of the filter paper changed from white to green and emitted obvious blue-green fluorescence under irradiation at 365 nm (Figure 3B(d)). These results further verify the feasibility of this study.

### 3.2. Selection of Fluorescent Signal

**Figure 4:**
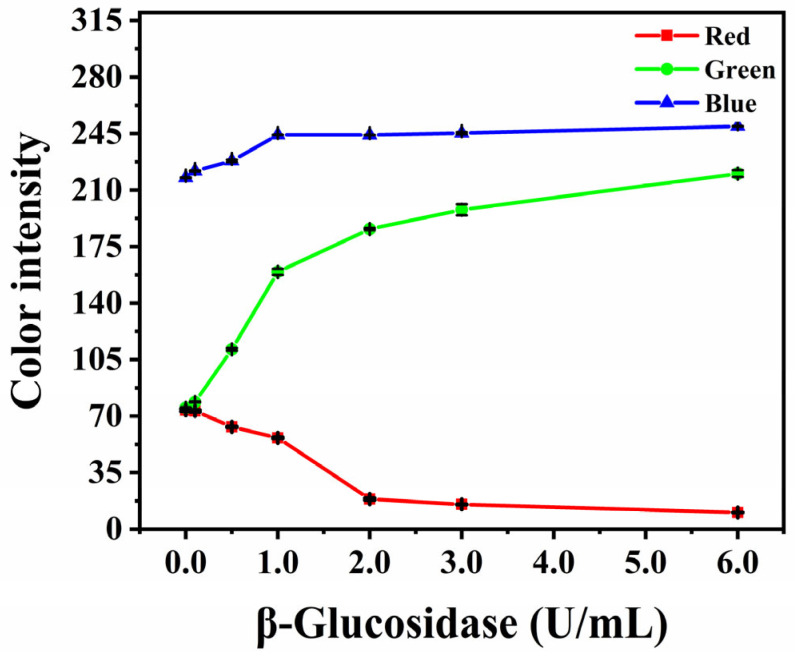
Intensity of RGB channels in fluorescent images irradiated by ultraviolet light at 365 nm of the paper-based device with different activity of β-glucosidase.

To analyze the fluorescent intensity of the detection area, a smartphone was used to take images of the paper-based device under the irradiation at 365 nm. Adobe Photoshop CC 2018 was used to process the image, and the RGB channel value was used to represent the fluorescent signal of the detection area. In order to obtain the optimal fluorescent signal, β-glucosidase solutions of different activities were added to the detection area, and the corresponding RGB channel values were obtained by Adobe Photoshop CC 2018. As shown in Figure 4, with the increase in β-glucosidase activity, the values of the blue and green channels increased, while the value of the red channel gradually decreased. Because UV irradiation is blue on filter paper, the value of blue channel increased slightly with the increase in β-glucosidase concentration. In order to make the fluorescent change as sensitively as possible, the value obtained through subtracting the red channel from the green channel was used to represent the fluorescent signal (Equation (1)). Additionally, the fluorescent signal difference between the test and blank samples is the relative fluorescent signal (Equation (2)).

### 3.3. Optimization of the Paper-Based Analytical Device

**Figure 5:**
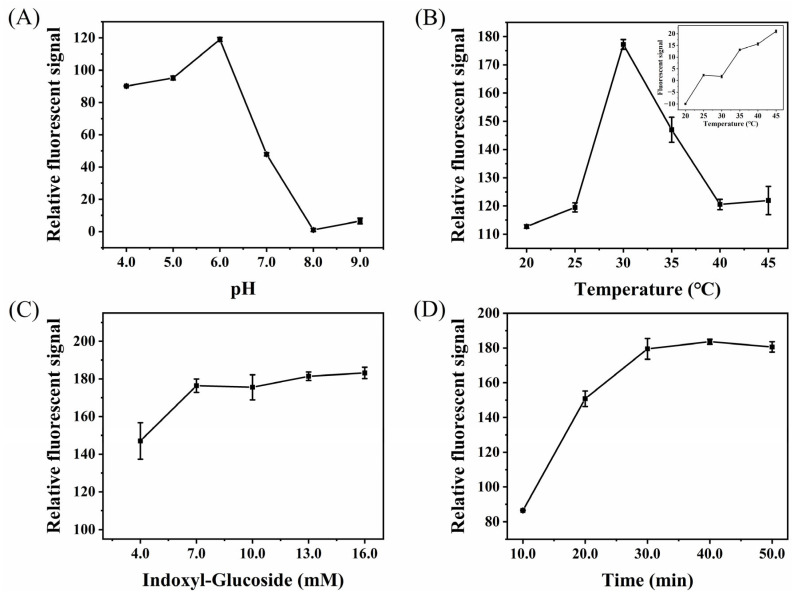
Effect of pH (**A**), temperature (**B**), indoxyl-glucoside concentration (**C**), and reaction time (**D**) on the fluorescent detection of β-glucosidase by the paper-based device, β-glucosidase = 1.0 U/mL. Inset: effect of temperature on the fluorescent detection of β-glucosidase by the paper-based device, β-glucosidase = 0.0 U/mL.

To obtain a high sensitivity of the paper-based device for detecting β-glucosidase, a series of parameters that may affect the analysis signal were optimized. In the optimization process, the relative fluorescent signal of the detection area was used as a marker, and the activity of β-glucosidase was 1.0 U/mL. The pH of the reaction system (buffer solution) that will affect the activity of β-glucosidase was first investigated. Phosphate buffered saline (PBS, 10 mM), with pH of 4.0–9.0, was prepared and used as the solvent of β-glucosidase for fluorescent detection. The results are shown in Figure 5A. From pH 4.0 to 6.0, the relative fluorescent signal gradually increased and reached the maximum at pH 6.0. However, with the further increase in pH, the relative fluorescent signal decreased sharply. Therefore, the PBS buffer solution of pH 6.0 was selected for the next study. The reaction temperature can not only affect the enzyme activity of β-glucosidase, but also affect the decomposition of substrate. Therefore, the change of relative fluorescent signal in the detection area was recorded when the reaction temperature varied from 20 °C to 45 °C. It can be seen from Figure 5B that, when the temperature is 30 °C, the relative fluorescent signal reaches the maximum value. The enzyme activity can be improved by increasing temperature, but too high temperature has an inhibition effect. In addition, with the increase in temperature, the substrate will be self-decomposed, thereby enhancing the fluorescent signal of the blank sample (Figure 5B inset). Thus, 30 °C was used as the reaction temperature. The higher the substrate concentration, the more products that may be generated under the same conditions. Therefore, the effect of substrate concentration (4.0–16.0 mM) on the relative fluorescent signal was investigated. It can be seen from Figure 5C that the relative fluorescent signal gradually increased with the increase in substrate concentration, and almost reached saturation when the substrate concentration was 7.0 mM, which was selected for the next study for a low analysis cost. Figure 5D shows the change of relative fluorescent signal with reaction time. After being reacted for 30.0 min, the relative fluorescent signal remained almost constant, indicating that the amount of generated fluorescent products reached the maximum at this time. Therefore, 30.0 min of reaction time was chosen for further study.

### 3.4. Analytical Performance of the Developed Paper-Based Analytical Device

Under the optimal conditions, the within- and between-batch repeatability, linear range, and limit of detection (LOD) of the paper-based device were studied to evaluate its analytical performance. The within-batch (*n* = 5) repeatability of the fluorescent and colorimetric detection of the paper-based device were 3.4% and 4.3%, and the between-batch (*n* = 5) repeatability were 0.8% and 2.0%, respectively, indicating a good reliability of the established method.

**Figure 6:**
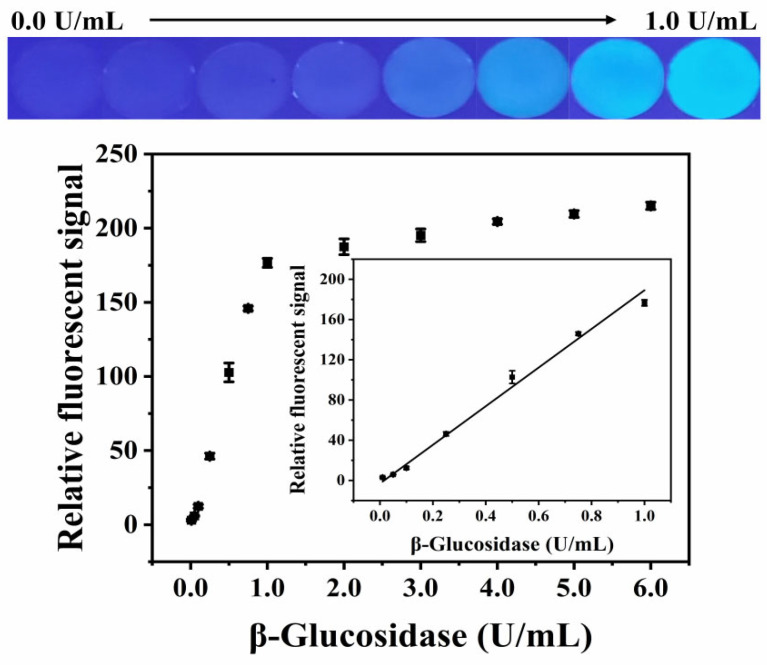
The relative fluorescent value of the paper-based device changed with the activity of β-glucosidase. Inset: calibration plot in the linear range of 0.01–1.00 U/mL. Photographs: fluorescent images of paper-based device at different β-glucosidase activity.

Figure 6 shows the relative fluorescent signal of the paper-based device versus the activity of β-glucosidase, in the range of 0.00–1.00 U/mL. It can be seen from the graphics that, with the increase in β-glucosidase activity, the detection area gradually emitted bright blue-green fluorescence, accompanied by an increase in the relative fluorescent signal. The relative fluorescent signal showed a good linear relationship with the β-glucosidase activity, in the range of 0.01–1.00 U/mL (Y = 192.1756 × C_[β-glucosidase]_ − 3.0873, R^2^ = 0.9924), and the LOD was 0.005 U/mL (LOD = 3 *σ*/*S*, where *σ* represents the standard deviation of 11 blank sample measurements, and *S* is the slope of the corresponding calibration plot).

**Figure 7:**
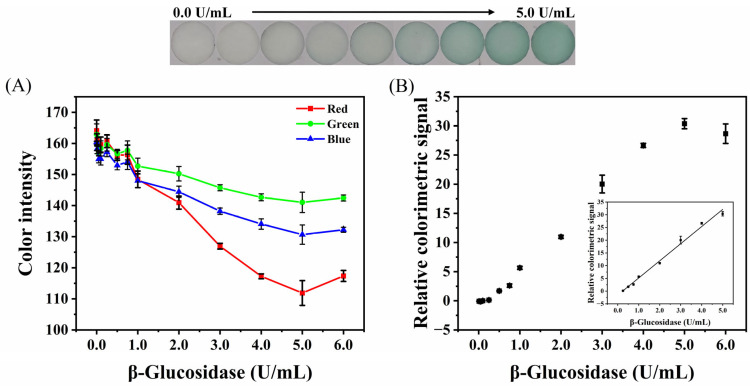
(**A**) Intensity of RGB channels in colorimetric images of the paper-based devices with different activity of β-glucosidase. (**B**) The relative colorimetric value of the paper-based device is changed with the activity of β-glucosidase. Inset: calibration plot in the linear range of 0.25–5.00 U/mL. Photographs: colorimetric images of paper-based device at different β-glucosidase activity.

The colorimetric method was also used to detect the activity of β-glucosidase. The RGB channel value of the obtained image is shown in Figure 7A. The values of the RGB channels decreased with the increase in β-glucosidase activity, and the change of the red channel was the most sensitive, followed by the blue and green channels. Therefore, the value obtained through subtracting the red channel from the green channel was selected as the colorimetric signal. The RGB channel values were processed by Equations (1) and (2) to obtain the relationship between the relative colorimetric signal and the activity of β-glucosidase (Figure 7B). With the increase in β-glucosidase activity, the detection area changed from white to green, and the relative colorimetric signal increased. The relative colorimetric signal and the activity of β-glucosidase presented excellent linear dependence, in the range of 0.25−5.00 U/mL. The regression equation is Y = 5.7351 × C_[β-glucosidase]_ − 1.5741 (R^2^ = 0.9942), and the LOD is 0.0668 U/mL.

The performance of the method in this study was compared with some reported methods for the detection of β-glucosidase (Table 1). Compared with other reports for fluorescent or colorimetric detection of β-glucosidase, this method does not use large equipment and synthesize complex materials, but has the advantages of simple operation and low cost. Furthermore, compared with the PGM or other paper-based analytical devices, this method expands on the detection range and lowers the LOD, which can realize the detection of β-glucosidase from low to high activity. Moreover, this dual-mode analysis method produces two detection signals that can verify the detection results with each other, making the results more reliable. To the best of our knowledge, this is the first time that the dual-mode detection of β-glucosidase has been achieved by fluorescence and colorimetry.

### 3.5. Specificity of the Paper-Based Analytical Device for β->Glucosidase Detection

**Figure 8:**
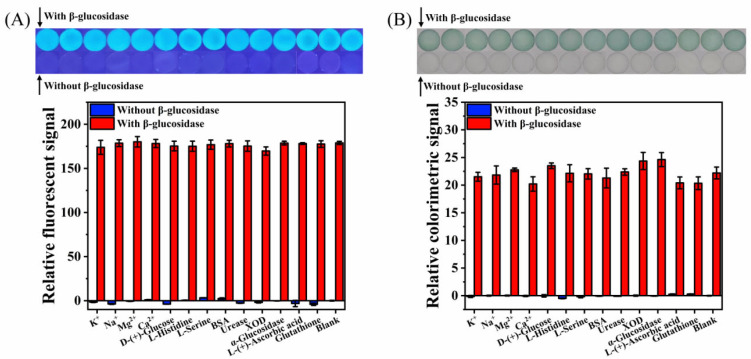
Selectivity and interference study of the paper-based device for β-glucosidase detection: (**A**) fluorescence, (**B**) colorimetry.

To evaluate the specificity of the designed paper-based device for the detection of β-glucosidase in complex matrices, the selectivity and interference study were performed. The response values of some substances that may exist in real samples on the paper-based device under the optimal conditions were recorded, such as metal ions (2.0 mM of Na^+^, K^+^, Ca^2+^, and Mg^2+^), D-(+)-glucose (2.0 mM), amino acids (0.1 mM of L-serine and L-histidine), BSA (0.1 mg/mL), reducing substances (0.01 mM of L-(+)-ascorbic acid and glutathione), and other enzymes (15.0 U/mL of urease, XOD, and α-glucosidase). The β-glucosidases used in fluorescent and colorimetric detection were 1.0 U/mL and 3.0 U/mL, respectively. As shown in Figure 8A,B, compared with the blank sample, the addition of interfering substances will not produce fluorescent or colored substances, and the presence of interfering substances will not cause significant interference on the fluorescent or colorimetric detection of β-glucosidase. These results show that the developed paper-based device has excellent selectivity for β-glucosidase detection. In addition, it should be noted that trace amounts of reducing substances (0.01 mM of L-(+)-ascorbic acid and glutathione) do not significantly affect the detection results (Figure 8). However, according to the detection principle, a large number of reducing substances will affect the indoxyl auto-oxidation process. Therefore, when there are a large number of reducing substances in the test sample, the influence of reducing substances on the detection results can be reduced by pretreatment of the sample. For example, ascorbic acid oxidase or glutathione peroxidase is added to remove the effect of ascorbic acid or glutathione on the detection results.

### 3.6. Real Samples Analysis

To evaluate the applicability of the developed method in real sample analysis, the β-glucosidase activities in spiked normal human serum and crude almond samples were measured. There was no β-glucosidase detected in the human serum sample, and the spiked recoveries of the fluorescent and colorimetric detection methods were 101.3−117.9% and 95.7−118.0%, respectively (Table 2). The β-glucosidase activities of the crude almond sample detected by the fluorescent and colorimetric detection methods were 23.62 ± 0.53 U/mL and 23.86 ± 0.25 U/mL, respectively, which are comparable results. The spiked recoveries of the crude almond samples, measured by the fluorescent and colorimetry, were 96.1−105.4% and 87.5−92.2%, respectively (Table 3). The recovery range was similar to other reported literatures [45,46,47]. These results show that this method can be used for the detection of β-glucosidase in actual samples with high reliability.

## 4. Conclusions

In this study, indoxyl-glucoside was deposited on filter paper to develop a paper-based analytical device for fluorescent and colorimetric dual-mode detection of β-glucosidase for the first time. Fluorescent and colorimetric images were captured using a smartphone, and the RGB channel values in the images varied with the activity of β-glucosidase. This method has excellent specificity and good spiked recoveries for the detection of β-glucosidase in real samples. The increase in the detection mode improves the reliability of the detection results and expands the application scope of detection. The developed paper-based device is simple, environmentally friendly, and cost-effective, which is a potential POCT device. In the future, the combination of an ultraviolet lamp and an LED strip can be designed as a camera obscura with a controllable light source mode, and the color processing software can be designed as smartphone application software, which can further improve the portability and user friendliness of the paper-based equipment. This small detection device has a good application prospect, especially in the areas that are lacking in resources.

## Figures and Tables

**Table 1:** Comparison of detection methods for β-glucosidase.

Detection Methods	Platform	Main Materials	Linear Range (U/mL)	LOD (U/L)	Ref.
Colorimetric analysis by UV–Vis spectrophotometer	Solution	Gold–cellobiose nanocomposites	0.003–0.100	1.0	[42]
Glucometer-based assay	Solution	D-(−)-Salicin	1.0–9.0	450	[20]
Glucometer-based assay	Solution	D-(−)-Salicin	0.0873–1.5498	-	[12]
Fluorescent analysis by spectrophotometer	Solution	Carbon dot, *p*-nitrophenol	0.005–0.250	12.3	[43]
Fluorescent analysis by spectrophotometer	Solution	*β*-arbutin, polyethylenimine molecules	0.001–0.036	0.4	[16]
Fluorescent analysis by spectrophotometer	Solution	CuInS_2_ quantum dots, Cu^2+^, amygdalin	0.0005–0.7000	0.2	[19]
Fluorescent analysis by spectrophotometer	Solution	BSA-Cu_3_(PO_4_)_2_·3H_2_O nanoflowers, Amplex Red, hydrogen peroxide, Amygdalin	0.0005–1.5000	0.33	[1]
Fluorescent analysis by commercial plate reader	Paper	Mono-*β*-glucoside derivative of 2,3-dihydoxynaphthalene, Tb-Cholate gel	-	76.2	[44]
Fluorescent analysis by smartphone	Paper	Indoxyl-Glucoside	0.01–1.00	5.0	This work
Colorimetric analysis by smartphone			0.25–5.00	66.8	

**Table 2:** Detection of β-glucosidase in normal human serum (*n = 3*).

Methods	Added (U/mL)	Total Found (U/mL)	Recovery (%)	RSD (%)
Fluorescence	0.00	- ^a^	-	-
	0.25	0.25 ± 0.02	101.3	7.3
	0.50	0.59 ± 0.01	117.9	2.0
	0.75	0.79 ± 0.01	105.9	1.1
Colorimetry	0.00	- ^a^	-	-
	1.00	1.12 ± 0.02	112.3	2.2
	2.00	2.36 ± 0.01	118.0	0.6
	3.00	2.87 ± 0.10	95.7	3.6

^a^ Not detected by this method.

**Table 3:** Detection of β-glucosidase in crude almond sample (*n = 3*).

Methods	Added (U/mL)	Total Found (U/mL)	Recovery (%)	RSD (%)
Fluorescence	0.0	0.18 ± 0.00	-	2.2
	0.3	0.49 ± 0.02	101.0	4.8
	0.5	0.86 ± 0.02	96.1	2.4
	0.7	0.92 ± 0.02	105.4	1.6
Colorimetry	0.0	0.37 ± 0.01	-	3.1
	1.0	1.29 ± 0.07	92.2	5.7
	2.0	2.12 ± 0.10	87.7	4.8
	3.0	2.99 ± 0.16	87.5	5.2

## Data Availability

Not applicable.
